# Prevalence and correlates of depressive symptoms among nurses during COVID-19 pandemic

**DOI:** 10.1186/s41983-023-00616-8

**Published:** 2023-01-30

**Authors:** Eman Ahmed Ali, Mohammad Gamal Sehlo, Ramadan Abdelbr Hussein, Eman Tarek Ali, Ahmed Mohamed Abdalla

**Affiliations:** Psychiatry Department, Faculty of Medicine, Zagazig University, P.O. Box 44519, Zagazig, Egypt

**Keywords:** COVID-19, Nurses, Depression

## Abstract

**Background:**

During any critical health care situation as COVID-19 pandemic, it is expected that the medical staff will be under a high level of stress. However, nurses specifically are under both physical and psychological pressure during this pandemic, with a risk of mental health problems, such as anxiety and depression. Accordingly, nurses exposed to patients with COVID-19 infection are expected to suffer from a high level of depressive symptoms. This cross-sectional study was applied on 456 nurses with age ranges from 18 to 60. They were divided into two groups: group 1 were composed of 228 nurses who were directly exposed to suspected or confirmed cases of COVID-19 patients, while group 2 were composed of 228 who were less exposed to suspected or confirmed cases of COVID-19 patients for comparison. Data were collected by personal interviews with nurses using Patient Health Questionnaire 9 (PHQ 9) scale for assessment of presence of depressive symptoms and its severity. We aimed to assess the both the prevalence and the predictors of depressive symptoms among nurses exposed to COVID-19 patients.

**Results:**

We found a statistically significant higher percentage of depressive symptoms among nurses directly exposed to COVID-19 patients (61.8%) versus the less exposed group (18%). There was a statistically and significantly higher specific COVID-19 associated stressors score (SCAS) among nurses directly exposed to suspected or confirmed cases of COVID-19 patients compared to those who were less exposed; likewise, the PHQ-9 score was a statistically and significantly higher among directly exposed group compared to less-exposed group. Moderate and severe depressive symptoms were present in 23.2% and 22.4%, respectively, within the group of nurses with direct exposure; meanwhile, the less-exposed group showed 7.5% and 3.9%, respectively, with statistically higher significant difference. On doing a linear regression analysis, all the following predictors were significantly independently associated with higher PHQ-9 scores (with higher depressive symptoms severity) among nurses exposed to COVID-19: physical isolation (restrictions on touching others, even after working hours), exposure to a new COVID-19 patient, developing COVID-19-like symptoms, displaying COVID-19-like symptoms by colleagues, knowing that COVID mortality rate exceeds influenza, possible separation from family, concern about family members, fears about infection for patients, family, and friends.

**Conclusions:**

COVID-19 pandemic has serious effects on the psychological well-being of nurses exposed to COVID-19 patients. There was an increased rate of depressive symptoms among them during the pandemic with its subsequent burden. Therefore, nurses exposed to COVID-19 patients are in a high need of care and support during the pandemic.

## Background

Specialized workplace issues such as excessive stress, overworked clinical job, and occupational stress pose a severe threat to nurses' mental health. Furthermore, nurses frequently encounter a variety of life experiences, including illness, trauma, and even death, which have additional physical and psychological impacts on them. In addition to having an immediate impact on their own health, nurses' psychological well-being has also an impact on the quality of medical care they deliver to the patients in a hospital setting [1].

For example, depressive symptoms can reduce the cognitive performance that can affect the work performance, resulting in a decreased work efficiency which may ultimately have an impact on the standard of medical care [2]. Coronavirus disease-2019 (COVID-19) is an infectious disease brought on by coronavirus 2 that causes severe acute respiratory syndrome (SARS-CoV-2). The condition was initially identified in December 2019 in Wuhan, China, then it has spread throughout the world [3]. The World Health Organization Emergency Committee declared COVID-19 as an international public health emergency and an outbreak in late January 2020 [4]. Since then, the world's health care systems are overburdened [5]. Due to the high risk of infection, lack of personal protection equipment, lack of appropriate treatment, feeling of being inadequately supported, loss of control, overwork, significant lifestyle changes, quarantine, and social isolation, healthcare workers including nurses are under a lot of pressure from COVID-19 pandemic [6]. Facing the critical situation of this growing pandemic, nurses are under both physical and psychological pressure; they are at risk of stress [7], and other mental health problems, such as anxiety and depression [8–10].

Al Amer et al. and Arafa et al., found that depression, anxiety, and stress are comorbidities that are highly prevalent among both nurses and those who contacted COVID-19 patients [11, 12]. Another study in Muscat reported that 18.1% of primary healthcare workers during the COVID-19 pandemic showed depressive symptoms [13].

Accordingly, the present study aimed to estimate the prevalence and correlates of depressive symptoms among nurses working at Zagazig University hospital in Egypt during COVID-19 pandemic. Knowing that nurses are a cornerstone in the health care system, it is an important issue to care for their psychological well-being to raise the quality of medical care.

## Methods

### Participants

A consecutive sample of 456 nurses were divided into two groups: group 1 (exposed group) was composed of 228 nurses who were directly exposed to suspected or confirmed cases of COVID-19 patients, and group 2 (less-exposed group) was composed of 228 who were less exposed to suspected or confirmed cases of COVID-19 patients. The sample size was calculated using open epi software. This cross-sectional study that was conducted from June 2021 to December 2021. The exposed group was recruited from the Isolation Department from inpatient (chest) wards and outpatient clinics (chest, ENT, and radiology); participants in this group were directly exposed to suspected or confirmed cases of COVID-19 patients, in Zagazig University Hospitals, Zagazig, Egypt. While the less-exposed group was recruited from Intensive Care Units, inpatient wards (Psychiatry, Rheumatology, Ophthalmology, Neurology, Tropical, Cardiology, Dermatology, Pediatric, Oncology, Surgery, and Orthopedic Departments) and outpatient clinics (Psychiatry, Rheumatology, Ophthalmology, Dermatology, Surgery, and Orthopedic Departments); participants in this group were less exposed to suspected or confirmed cases of COVID-19 patients, in Zagazig University Hospitals, Zagazig, Egypt. The data were collected by the study team. Eligibility of subjects for participation in the study was determined according to the following specified inclusion and exclusion criteria: the inclusion criteria: nurses who are currently working at Zagazig University Hospitals in a full-time work for at least 1 year of either sex, and age ranges from 18 to 60. The exclusion criteria: past history of any psychiatric disorder or substance abuse, or presence of any severe medical illness that could affect psychological condition (as cancer or autoimmune disorders). The exclusion was done by history taking and revision of the medical files of the nurses.

### Measures

1. Sociodemographic form that is composed of questions related to personal characteristics of the nurses including age, gender, marital status, education, residence, years of work, and smoking status.
2. PHQ 9 (Patient Health Questionnaire 9):
  The Patient Health Questionnaire (PHQ 9) was used to assess depressive symptoms [14]. The PHQ 9 is a widely used measure for identifying depressive symptoms, diagnosing depressive disorder; it has excellent psychometric properties when used in medical and psychiatric patients. The PHQ 9 evaluates the presence and severity of the nine primary symptoms of major depression in accordance with the DSM-IV diagnostic criteria for major depressive disorder. This makes it possible to determine both the presence of depressive disorder and its degree as well. Scores of 5, 10, 15, and 20 correspond to mild, moderate, moderately severe, and severe depression [15]. A validated Arabic version of the scale was used in this study. The reliability test was done with Cronbach’s Alpha equals 0.857 [16].
3. Specific COVID-19 Associated Stressors Score (SCAS) questionnaire:
  It is derived and modified from the US National Center for Posttraumatic Stress Disorder 2020 and MERS-CoV staff questionnaire [17]. It consisted of 19 items on 5-point Likert scale with 0 = never exposed, 1 = never stressful, 2 = occasionally stressful, 3 = frequently stressful, and 4 = extremely stressful; total scores could range from 0 to 76. The items are grouped into four subscales as the following: the need to employ strict biosecurity measures, exposure to infection risk, personal demands and fears, and stigma. For each subscale, the participants’ responses on each item are scored and summed to obtain a total score; higher scores indicate higher stress levels. The level of Specific COVID-19 Associated Stress Score was calculated by summing the total score of all subscales; consequently, they were classified as the following: low (score ≤ 25), moderate (26–50), and high (51–76) [18]. The Arabic version used in this study was translated and used in a previous study. The reliability test was done with Cronbach’s Alpha equals 0.87 [18].

### Statistical analysis

The data analysis and sample size calculations (with 80% power) were performed using the statistical package for social sciences (SPSS version 20). The categorical data were presented in the form of number and percentage with Chi square was used as a test of significance. Continuous data were expressed as mean ± SD (Standard deviation) and Median with the Interquartile range (IQR). The Independent *T* test and Mann Whitney test were used as a test of significance for the differences among groups. Multiple regression analysis was used to assess the predictors of depression. We tested the normality of the data regarding the sample size using Kolmogorov–Smirnov (K–S) test with a *p* value > 0.05 that proves the normality. A *p* value < 0.05 was used to indicate statistical significance.

## Results

We found a statistically significant higher percentage of depression among nurses directly exposed to COVID-19 patients (61.8%) versus those not directly exposed (18%).

Table 1 shows that the mean age of nurses directly exposed to COVID-19 and those not directly exposed to COVID-19 was 30.24 and 30.69 years, respectively, with non-significant statistical difference. Females represented 60.1% and 67.5% of nurses directly exposed to COVID-19 and those less-directly exposed to COVID-19, respectively, with non-significant statistical difference. Rural residents represented 75% and 77.2% of nurses directly exposed to COVID-19 and those less-directly exposed to COVID-19, respectively, with statistically non-significant difference. Institute level of education represented 78.1% and 74.1% of nurses directly exposed to COVID-19 and those not directly exposed to COVID-19, respectively, with statistically non-significant difference. Married nurses represented 58.3% and 62.7% of directly exposed to COVID-19 and those less-directly exposed to COVID-19, respectively, with statistically non-significant difference. Smokers represented 8.3% and 6.6% of nurses directly exposed to COVID-19 and those not directly exposed to COVID-19, respectively, with statistically non-significant difference. There was non-significant difference between both groups regarding years of work (median years were 16 and 15 years in directly less-directly exposed, respectively).

**Table 1:** Comparison between the studied groups regarding demographic data and smoking

Parameter	Groups		Test	
	Nurses directly exposed to COVID-19 group	Nurses less exposed to COVID-19 group	Test of sig	*p*
	*N* = 228 (%)	*N* = 228 (%)		
Age (year)				
Mean ± SD	30.24 ± 9.04	30.69 ± 8.53	*t* − 0.549	0.583
Years of work				
Median (range)	16 (5–37)	15 (4–32)	Z − 0.002	0.999
Gender				
Male	91 (39.9%)	74 (32.5%)	χ^2^ 2.745	0.098
Female	137 (60.1%)	154 (67.5%)		
Residence				
Rural	171 (75%)	176 (77.2%)	χ^2^ 0.301	0.583
Urban	57 (25%)	52 (22.8%)		
Marital status				
Single	91 (39.9%)	79 (34.6%)	χ^2^ 2.108	0.146
Married	133 (58.3%)	143 (62.7%)		
Divorced	2 (0.9%)	0 (0%)		
Widowed	2 (0.9%)	6 (2.6%)		
Education				
Institute	178 (78.1%)	169 (74.1%)	χ^2^ 0.977	0.323
College	50 (21.9%)	59 (25.9%)		
Site				
Outpatient	59 (25.9%)	78 (34.2%)	χ^2^ 2.095	0.148
Inpatient	85 (37.3%)	72 (31.6%)		
ICU	84 (36.8%)	78 (34.2%)		
Smoking				
No	209 (91.7%)	213 (93.4%)	χ^2^ 0.509	0.476
Yes	19 (8.3%)	15 (6.6%)		

χ^2^ Chi square test t independent sample t test *Z* Mann Whitney test

Table 2 shows a statistically significant higher Specific COVID-19 Associated Stressors Scores (SCAS) among nurses directly exposed to suspected or confirmed cases of COVID-19 patients compared to less-exposed group. Moderate and severe (SCAS) were present in 36% and 23.7%, respectively, within the exposed group versus 22.8% and 10.1% within less-directly exposed group with high statistically significant difference.

**Table 2:** Comparison between the studied groups regarding Specific COVID-19 Associated Stressors (SCAS)questionnaire score

Parameter	Groups		Test	
	Nurses directly exposed to COVID-19 group	Nurses less exposed to COVID-19 group	Test of sig	*p*
	*N* = 228 (%)	*N* = 228 (%)		
SCAS score				
Median	52	31	Z − 15.721	< 0.001**
IQR	44–59	1–38		
Range	19–71	2–49		
SCAS				
Low	92 (40.3%)	153 (67.1%)	χ^2^ 203.421	< 0.001**
Moderate	82 (36%)	52 (22.8%)		
High	54 (23.7%)	23 (10.1%)		

*Z* Mann Whitney test χ^2^ Chi square for trend test **p ≤ 0.001 is statistically highly significant IQR interquartile range

Table 3 shows that PHQ-9 score was significantly higher among directly exposed group compared to less-exposed group. Moderate and severe depression were present in 23.2% and 22.4%, respectively, within the exposed group versus 7.5% and 3.9% within less-directly exposed group with high statistically significant difference.

**Table 3:** Comparison between the studied groups regarding Patient Health Questionnaire (PHQ) score

Parameter	Groups		Test	
	Nurses directly exposed to COVID-19 group	Nurses less exposed to COVID-19 group	Test of sig	*p*
	*N* = 228 (%)	*N* = 228 (%)		
PHQ-9 score				
Median	12	9	*Z* (− 5.745)	< 0.001**
IQR	9–17	6–13		
Range	1–26	0–22		
PHQ-9				
No depression	87 (38.2%)	187 (82%)	χ^2^ 84.495^§^	< 0.001**
Mild depression	37 (16.2%)	15 (6.6%)		
Moderate depression	53 (23.2%)	17 (7.5%)		
Severe depression	51 (22.4%)	9 (3.9%)		

*Z* Mann Whitney test χ^2^ Chi square for trend test **p ≤ 0.001 is statistically highly significant IQR interquartile range

On doing a linear regression analysis, all the following variables were significantly independently associated with higher PHQ-9 scores (with higher depression severity) among nurses exposed to COVID-19: physical isolation (restrictions on touching others, even after working hours) (*β* = 3.34, *p = *0.02), every time being exposed to a new COVID-19 patient (*β* = 5.56, *p = *0.009), developing COVID-19-like symptoms (*β* = 7.45, *p = *0.006), colleagues displaying COVID-19-like symptoms (*β* = 6.14, *p = *0.007), a relatively higher mortality rate compared to influenza (*β* = 3.02, *p = *0.03), possible separation from family, and concern about family members (*β* = 7.02, *p = *0.007), and fears about infection for patients, family and friends (*β* = 7.99, *p = *0.006) (Table 4).

**Table 4:** Linear regression analysis of factors independently associated with PHQ-9 score among nurses exposed to COVID-19

	β	*t*	*P*	CI (95%)
Age	− 0.41	1.77	0.08	− 66 to 1.23
Female gender	0.39	1.68	0.09	− 0.10 to 3.46
Divorced	0.44	1.80	0.1	− 0.54 to 3.32
Physical strain of protective equipment (dehydration, heat, exhaustion)	0.88	1.90	0.2	− 0.97 to 6.99
Physical isolation (restrictions on touching others, even after working hours)	3.34	4.135	0.02*	1.77 to 11.07
Constant awareness and vigilance regarding infection control procedures	0.63	1.90	0.1	− 0.31 to 4.98
Pressures regarding procedures that must be followed (lack of spontaneity)	0.28	1.11	0.3	− 0.11 to 2.90
Every time being exposed to a new COVID-19 patient	5.56	8.65	0.009*	2.87 to 14.67
Getting screened for COVID-19 infection after exposure	0.12	1.01	0.4	− 0.09 to 1.58
Developing COVID-19-like symptoms	7.45	9.14	0.006*	4.89 to 21.66
Colleagues displaying COVID-19-like symptoms	6.14	6.55	0.007*	3.77 to 19.55
The extended symptom-free incubation period of COVID-19	0.57	1.89	0.1	− 0.22 to 3.44
A relatively higher mortality rate compared to influenza	3.02	3.11	0.03*	1.08 to 9.87
Possible separation from and concern about family members	7.02	8.60	0.007*	3.99 to 18.66
Fears about infection for patients, family and friends	7.99	9.11	0.006*	4.22 to 28.90
Not knowing when the COVID-19 outbreak will be under control	0.98	1.99	0.1	− 0.88 to 4.11
Lack of treatment for COVID-19	0.56	1.11	0.2	− 0.41 to 3.77
News of new cases of COVID-19 reported in TV/newspaper	0.16	1.09	0.3	− 0.13 to 2.98
Shortage of staff or adequate protective measures at times	0.66	1.17	0.2	− 0.16 to 4.65
Inner conflict between duty and own safety	0.44	1.10	0.3	− 0.14 to 3.99
Others’ fear of contact with those treating patients with COVID-19	0.89	1.95	0.2	− 0.33 to 5.11
Developing self-stigma about voicing needs and fears	0.34	1.66	0.3	− 0.22 to 3.08

*p < 0.05 is statistically significant

## Discussion

In our study, we found statistically significant higher percentage of depression among nurses directly exposed to COVID-19 patients (61.8%) versus less-exposed group (18%). In addition, mild, moderate, and severe depression among directly exposed group were 16.2%, 23.2%, 22.4% versus 6.6%, 7.5%, 3.9% in less-exposed group, respectively, with a statistically significant higher difference. Few studies have investigated depression among exposed and less exposed nurses, and most of them were consistent with our results about the increased prevalence of depression among the directly exposed group; nevertheless, there may be varying percentages. This could be explained by the severe stress of the COVID-19 pandemic suffered by the nurses. In line with our results, Cai et al. showed a statistically significant higher depression level in directly exposed nurses versus less exposed nurses during the outbreak period; moderate to severe depression represented 21.8% in directly exposed nurses versus 9.1% in less exposed [19]. Another similar result was obtained in a study by Gilleen et al. who reported a significant higher depression among directly exposed versus less exposed health care workers (61.19% versus 52.49%, respectively); severe depression represented (6.37% versus 3.84%, respectively) [20]. Furthermore, two studies from Italy by Trumello et al., and Heidarijamebozorgi et al. reported a statistically significant increase in the prevalence of depression among health care workers directly exposed to COVID-19 patients versus less exposed with a more severe depression among the directly exposed group [21, 22]. Another study in China by Lai et al. showed a statistically significant higher depression rate in health care workers directly exposed to COVID-19 patients (58.4%) versus less exposed (44.6%); moderate symptoms of depression represented (11.3%) versus (6.6%), respectively [8]. In addition, Cai et al. found that directly exposed medical workers had a higher rate of depressive symptoms (14.3%) versus (10.1%) in less exposed workers [23]. In addition, another study in Ethiopia by Mulatu et al. reported a statistically significant higher depression in health care workers directly exposed to COVID-19 patients (23.6%) versus less exposed group (17.6%) [24]. Finally, this agreed with two studies done in Egypt which found (93%) and (77.2%) of directly exposed health care workers had depressive symptoms [25, 26]. Using linear regression analysis to assess the predictors of depressive symptoms among the directly exposed group, all the following variables were significantly independently associated with increased severity of depression among nurses exposed to COVID-19, physical isolation (restrictions on touching others, even after working hours), exposure to a new COVID-19 patient, developing COVID-19-like symptoms, displaying COVID-19-like symptoms by colleagues, knowing that COVID mortality rate exceeds influenza, possible separation from family, concern about family members, fears about infection for patients, family, and friends. Our regression analysis model results were consistent with other studies like one by Dziedzic et al. who found that frequent contact with patients suspected of being infected with COVID-19 was a predictor of depressive symptoms among nurses exposed to COVID-19 patients [27]. In addition, Xiao et al. found in their regression analysis model that health care workers who had contact with COVID-19 patients were twice likely to develop depression [28]. Moreover, Chew et al. found that the physical symptoms of COVID-19 infection (cough, sore throat, breathlessness, lethargy, myalgia) increase the severity of depressive symptoms among nurses, while Saeed et al. found that a colleague diagnosed with COVID-19 infection emerged as a significant risk factor for depression [29, 30]. Furthermore, Baraka et al. reported that the presence of infected colleagues was significant predictor for depression; meanwhile, Elgohary et al. found that health care workers who had fears of COVID-19 infection for themselves or relatives were four times more likely to have depression [25, 31]. Luceño-Moreno et al., and Zheng et al., found that the fear of infection was associated with a higher risk of depressive symptoms [32, 33].

All these predictors for depressive symptoms in directly exposed nurses to patients with COVID 19 during the pandemic should be taken into account when developing a supportive program to decrease the burden of the depressive symptoms among those nurses.

## Conclusions

Our study revealed a high prevalence of depression among nurses exposed to suspected or confirmed cases of COVID-19 patients. All the following predictors were significantly independently associated with increased risk of depression: physical isolation (restrictions on touching others, even after working hours), exposure to a new COVID-19 patient, developing COVID-19-like symptoms, displaying COVID-19-like symptoms by colleagues, knowing the higher mortality rate of COVID compared to influenza, possible separation from, concern about family members and fears about infection for patients, family, and friends. Therefore, implementation of targeted interventions aiming to enhance the psychological support of nurses exposed to COVID-19 patients with reduction of their stressors are mandatory; this will result in not only on the improvement of depression among those nurses but also a better proper care for the patients.

### Limitations of the study

Our study has certain limitations, because exposures and outcomes were assessed simultaneously in a cross-sectional study; longitudinal investigations are advised, because there is no evidence of a causal relationship between exposure and outcome. However, we have many strengths in our study such as focusing on nurses exposed to patients with COVID-19 infection who are one of the highly important sectors among the health system in Egypt, and they were already under severe stress. Our study was performed by direct interview, not online questionnaires, which guarantees correct understanding of the patients to the questions and good interpretation of the results.

## Data Availability

Available upon request.

## Abbreviations

COVID-19: Coronavirus disease of 2019
PHQ 9: Patient Health Questionnaire 9
SCAS: Specific COVID-19 associated stressors questionnaire
